# Developing a New Two-Step Protocol to Generate Functional Hepatocytes from Wharton's Jelly-Derived Mesenchymal Stem Cells under Hypoxic Condition

**DOI:** 10.1155/2013/762196

**Published:** 2013-05-30

**Authors:** Patcharee Prasajak, Wilairat Leeanansaksiri

**Affiliations:** ^1^Stem Cell Therapy and Transplantation Research Group, Suranaree University of Technology, Nakhon Ratchasima 30000, Thailand; ^2^School of Microbiology, Institute of Science, Suranaree University of Technology, Nakhon Ratchasima 30000, Thailand

## Abstract

The shortage of donor livers and hepatocytes is a major limitation of liver transplantation. Thus, generation of hepatocyte-like cells may provide alternative choice for therapeutic applications. In this study, we developed a new method to establish hepatocytes from Wharton's jelly-derived mesenchymal stem cells (WJ-MSCs) cell lines named WJMSCs-SUT1 and WJMSCs-SUT2 under hypoxic condition. This new method could rapidly drive both WJ-MSCs cell lines into hepatic lineage within 18 days. The achievement of hepatogenic differentiation was confirmed by the characterization of both phenotypes and functions. More than 80% MSCs-derived hepatocyte-like cells (MSCDHCs) achieved functional hepatocytes including hepatic marker expressions both at gene and protein levels, glycogen storage, low-density lipoprotein uptake, urea production, and albumin secretion. This study highlights the establishment of new hepatogenic induction protocol under hypoxic condition in order to mimic hypoxic microenvironment in typical cell physiology. In conclusion, we present a simple, high-efficiency, and time saving protocol for the generation of functional hepatocyte-like cells from WJ-MSCs in hypoxic condition. The achievement of this method may overcome the limitation of donor hepatocytes and provides a new avenue for therapeutic value in cell-based therapy for life-threatening liver diseases, regenerative medicine, toxicity testing for pharmacological drug screening, and other medical related applications.

## 1. Introduction

Orthotopic liver transplantation has been shown to be an effective treatment for patients with end stage of liver dysfunction. However, this treatment is limited by the shortage of donor organs. Although hepatocytes transplantation has been shown to be successful treatment in some conditions such as liver-based metabolic disorders, the insufficient donor organs and hepatocytes remain obstacles for this technique [1]. Recently, stem cells are a promising tool for using as cell-based therapy because of their superior properties including self-renewal and broad differentiation potential into several cell types. To date, mesenchymal stem cells (MSCs) have been shown to obtain promising capacity not only multilineages differentiation potential but also immunomodulatory properties [2]. In addition, MSCs can be extensively expanded *in vitro*, due to effective cryopreservation and being easy to access from various sources such as bone marrow, adipose tissue, amniotic fluid, umbilical cord Wharton's jelly, and placenta [3–6]. These make MSCs become a good stem cell candidate for therapeutic purpose in clinical applications. 

Umbilical cord Wharton's jelly is an enriched source of MSCs which has superior advantages over the other sources such as noninvasive collection, less ethical concern, and enrichment with MSCs [7]. Several studies have demonstrated that MSCs from various sources including Wharton's jelly-derived mesenchymal stem cells (WJ-MSCs) possess hepatogenic differentiation potential both *in vitro* and *in vivo* [8–13]. Interestingly, hepatocyte-like cells generated from adipose tissue-derived MSCs showed therapeutic effect on mice models with both acute liver failure and chronic liver injury [14, 15]. Recent study also reported clinical improvement in patients with end-stage liver failure by chronic hepatitis C after transplantation with bone marrow-derived hepatocyte-like cells [16]. Based on these data, generation of hepatocyte-like cells from MSCs shows great potential in clinical use as regenerative medicine. 

According to previous published protocols, several studies have shown expensive cost, time consuming, and multiple steps induction of MSCs into hepatic lineage [10, 17–21]. Therefore, a simpler method is needed for developing an effective protocol to generate functional hepatocyte-like cells from MSCs. In this study, we developed a new method to induce WJ-MSCs cell lines, WJMSCs-SUT1, and WJMSCs-SUT2, into hepatic lineage followed by characterization of the MSCs-derived hepatocyte-like cells (MSCDHCs) at both cellular and molecular levels. Here, we show the achievement of hepatogenic differentiation of WJ-MSCs by using our new induction protocol under hypoxic condition. The hepatocyte-like cells generated from WJ-MSCs offer an alternative source of functional hepatocytes which will provide great advantages in liver disease treatments, drug discovery including toxicological research, and other medical applications.

## 2. Materials and Methods

### 2.1. Cell Lines

Two human WJ-MSCs cell lines, WJMSCs-SUT1 and WJMSCs-SUT2, were established and well characterized by Dr. Wilairat Leeanansaksiri's laboratory (Suranaree University of Technology, Thailand). WJMSCs-SUT1 and WJMSCs-SUT2 were derived from the cultivation of WJ-MSCs in Dulbecco's modified Eagle's medium with 1.0 g/L glucose (DMEM-LG) (Invitrogen, Carlsbad, CA, USA) containing 10% fetal bovine serum (FBS) (HyClone, Logan, UT, USA) and embryonic stem cells conditioned medium (ESCM) at 37°C with 5% CO_2_ and 5% O_2_, respectively. Both cell lines express cell surface markers: CD29^+^, CD44^+^, CD90^+^, CD34^−^, and CD45^−^, and contain capacity of differentiation into osteocytes, chondrocytes, and adipocytes as standard characteristics of mesenchymal stem cells.

### 2.2. Induction of Hepatogenic Differentiation *In Vitro*

**Figure 1:**
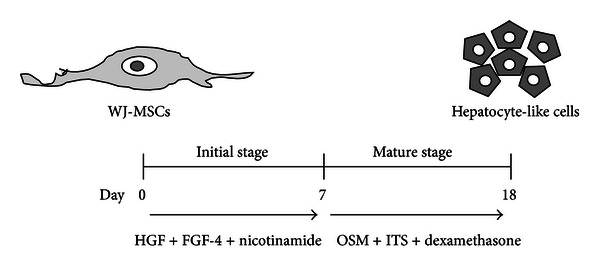
New method for hepatocyte-like cells generation from WJ-MSCs under hypoxic condition. This schematic figure represents two-step protocol for *in vitro* hepatogenic differentiation. In the first stage of induction, WJMSCs-SUT1 and WJMSCs-SUT2 were treated with the combination of HGF (20 ng/mL) + FGF-4 (10 ng/mL) + nicotinamide (5 mM) in DMEM serum-free medium for 7 days. To induce maturation, these cells were cultured in differentiation medium containing OSM (40 ng/mL) + ITS (20 *μ*L/mL) + dexamethasone (2 *μ*M) for a further 11 days.

The WJMSCs-SUT1 and WJMSCs-SUT2 were expanded until reaching 90% confluence and then subjected into hepatocytes differentiation processes by using our new two-step protocol (Figure 1). Briefly, the WJMSCs-SUT1 and WJMSCs-SUT2 were separately incubated in stage 1 differentiation medium containing serum-free DMEM-LG supplemented with 100 U/mL penicillin (Sigma, St. Louis, MO, USA), 100 *μ*g/mL streptomycin (Sigma, St. Louis, MO, USA), 2 *μ*g/mL amphotericin B (Bristol-Myers Squibb, NY, USA), 20 ng/mL human hepatocyte growth factor (HGF) (Peprotech, Rocky Hill, NJ, USA), 10 ng/mL fibroblast growth factor 4 (FGF-4) (Peprotech, Rocky Hill, NJ, USA), and 5 mM nicotinamide (Sigma, St. Louis, MO, USA) for 7 days. Subsequently, these cells were further cultured in stage 2 differentiation medium to achieve hepatic maturation which had components as serum-free DMEM-LG supplemented with 100 U/mL penicillin (Sigma, St. Louis, MO, USA); 100 *μ*g/mL streptomycin (Sigma, St. Louis, MO, USA); 2 *μ*g/mL amphotericin B (Bristol-Myers Squibb, NY, USA); 40 ng/mL oncostatin M (OSM) (Sigma, St. Louis, MO, USA); 2 *μ*M dexamethasone (Sigma, St. Louis, MO, USA), and 20 *μ*l/mL insulin, transferrin, and selenium (ITS + premix) (BD Biosciences, Franklin Lakes, NJ, USA) for up to 18 days. The differentiation medium was changed twice a week. The undifferentiated cells from both 2 cell lines were included as negative controls. 

### 2.3. Immunofluorescence Staining

Cells were fixed with 4% paraformaldehyde for 10 minutes at room temperature and permeabilized with 0.3% Triton X-100 in phosphate-buffered saline (PBS) for 20 minutes. Nonspecific immunostaining was prevented by 30 minutes incubation of the cells in PBS solution containing 3% bovine serum albumin (BSA) (Invitrogen, Carlsbad, CA, USA) at room temperature. Cells were then incubated in blocking solution overnight at 4°C with primary antibodies as follows: rabbit polyclonal anti-human albumin (ALB) and mouse monoclonal anti-human cytokeratin 18 (CK-18) (all 1 : 100) (DakoCytomation, Glostrup, Denmark). Cells were washed 3 times with PBS and incubated for 1 hour at room temperature with the secondary antibodies FITC-coupled polyclonal goat anti-mouse immunoglobulin G (1 : 100) (BD Biosciences, Franklin Lakes, NJ, USA) or FITC-coupled polyclonal swine anti-rabbit immunoglobulin G (1 : 100) (DakoCytomation, Glostrup, Denmark). Nuclei was revealed by 3 minutes of staining with the nuclear dye 4′,6-diamidino-2-phenylindole (DAPI) (Invitrogen, Carlsbad, CA, USA). After 3 washes, cells were mounted with antifading solution (Vector Laboratories, Burlingame, CA, USA) and examined under fluorescence microscope (BX51) (Olympus, Japan).

### 2.4. Periodic Acid-Schiff (PAS) Staining

After 4% paraformaldehyde fixation, cells were incubated for 5 minutes in 1% periodic acid (Sigma, St. Louis, MO, USA) and washed with distilled water prior to incubation with Schiff's reagent (Sigma, St. Louis, MO, USA) for 15 minutes. After 5-minute wash in tap water, hematoxylin counterstain was performed for 1 minute. Cells were washed and visualized under light microscope (CKX41) (Olympus, Japan). 

### 2.5. Uptake of Low-Density Lipoprotein Assay

The uptake of low-density lipoprotein (LDL) was detected with the Dil-Ac-LDL staining kit (Biomedical Technologies, Stoughton, MA, USA). The assay was performed according to the manufacturer's instructions. Briefly, cells were incubated in serum-free DMEM-LG containing 10 *μ*g/mL 1,1′-Dioctadecyl-3,3,3′,3′-tetramethylindocarbocyanine perchlorate-acetylated-LDL (Dil-Ac-LDL) for 4 hours at 37°C. Cells were then washed and visualized under fluorescence microscope (BX51) (Olympus, Japan).

### 2.6. Urea Assay

Cells were incubated with serum-free DMEM-LG containing 5 mM NH_4_Cl (Sigma, St. Louis, MO, USA) for 24 hours. Urea concentrations in supernatants were measured by colorimetric assay as QuantiChrom Urea Assay Kit (BioAssay Systems, Hayward, CA, USA) according to the manufacturer's instructions with an absorbance reader (Benchmark Plus) (Bio-Rad Laboratories, Hercules, CA, USA). Undifferentiated cells (day 0) and human hepatocellular carcinoma cell line (HepG2) were represented as a negative control and positive control, respectively.

### 2.7. Albumin Assay

After accomplishment of the differentiation, albumin concentrations in supernatants were screened by ELISA assay as AssayMax Human Albumin ELISA Kit (Assaypro, St. Charles, MO, USA) according to the manufacturer's instructions with an absorbance reader (Benchmark Plus) (Bio-Rad Laboratories, Hercules, CA, USA). Cell culture supernatants from undifferentiated cells (day 0) and HepG2 were represented as a negative control and positive control, respectively.

### 2.8. RNA Extraction and RT-PCR

Cells were subjected to total RNA extraction by using Total RNA Mini Kit (Tissue) (Geneaid, Taiwan) and RNase inhibitor treatment (Invitrogen, Carlsbad, CA, USA) according to the manufacturer's protocol. The cDNA was generated by RevertAid First Stand cDNA Synthesis Kit (Fermentas, St. Leon-Rot, Germany) according to the manufacturer's protocol. The corresponding cDNA was added into PCR master mix containing 10X PCR buffer, 1 U Taq polymerase, 25 mmol/L MgCl_2_, 10 mmol/L dNTP mixed, and 10 *μ*mol/L of each primer set for the corresponding target gene. The used primer sequences were shown in Table 1. Amplification conditions were as follows: initial denaturation at 95°C for 5 minutes followed by 35 cycles of denaturation at 95°C for 45 seconds, annealing at 56–62°C for 30 seconds (see Table 1 that refers to temperatures used), extension for 1 minute at 72°C, and a final extension at 72°C for 10 minutes. The samples were separated on a 2% agarose gel, stained with ethidium bromide, and photographed under UV light. Undifferentiated cells and HepG2 were represented as a negative control and positive control, respectively.

### 2.9. Statistical Analysis

All data were represented as mean ± standard deviation (SD) calculation. Statistical analysis was calculated by statistical software SPSS17.0 (SPSS Inc., Chicago, IL, USA) and results were analyzed by Student's *t*-test with significance at *P* < 0.05.

## 3. Results 

### 3.1. Morphology of MSCs-Derived Hepatocyte-Like Cells (MSCDHCs)

**Figure 2:**
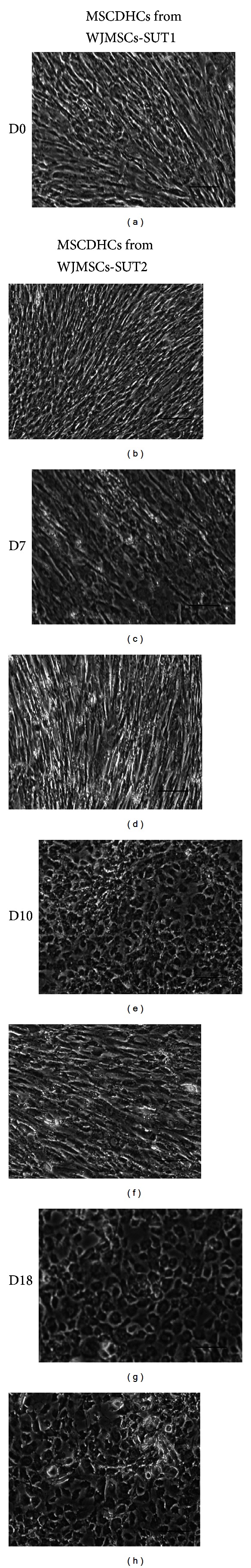
Hepatocyte-like cells feature of the differentiated cells during hepatogenic induction for 18 days. Morphological changing of MSCDHCs generated from WJMSCs-SUT1 ((a), (c), (e), and (g)) and WJMSCs-SUT2 ((b), (d), (f), and (h)) were observed following the differentiation period. Original magnifications: ×100 for all pictures. Bars indicate 100 *μ*m.

Morphological changing of the cells was determined on day 0, 7, 10, and 18 following differentiation. Upon induction, we observed morphological changing from fibroblastic cells of WJ-MSCs into polygonal round cells of hepatocytes feature. More than 80% of both WJMSCs-SUT1 and WJMSCs-SUT2 could be induced into hepatocyte-like cells morphology at the end of induction. Moreover, WJMSCs-SUT1 changed morphology from fibroblastic shape into epithelial round shape earlier on day 10 of differentiation whereas WJMSCs-SUT2 showed this feature on day 12 (Figure 2). For the control, both 2 cell lines did not show spontaneous differentiation into hepatocyte-like cells during the culture periods (data not shown). 

### 3.2. Expression of Hepatic-Lineage Markers

**Figure 3:**
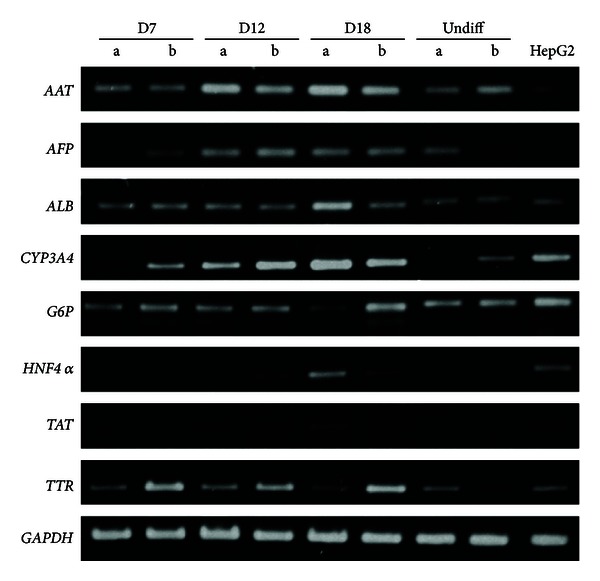
Hepatic-specific gene expressions of the differentiated cells after induction for 18 days. Total RNA was isolated from MSCDHCs generated from WJMSCs-SUT1 (lane a) and WJMSCs-SUT2 (lane b) following the differentiation period, and mRNA expressions of hepatic-specific genes were analyzed by RT-PCR. HepG2 was used as positive control. *GAPDH* was used as an internal control.

The mRNA expressions of hepatic-lineage markers were determined at different time points during differentiation periods. We found that undifferentiated stage of WJMSCs-SUT1 and WJMSCs-SUT2 could express low level of some hepatic-specific genes transcripts including *AAT*, *ALB*, *CYP*3*A*4, and *G*6*P* (Figure 3). Upon induction, MSCDHCs generated from both 2 cell lines showed the significant upregulation of mature hepatic markers, *AAT* and *CYP*3*A*4, in a time-dependent manner. Additionally, we observed correlation between the expressions of key hepatic transcription factors, *HNF*4*α*, and mature hepatic marker *ALB *in MSCDHCs generated from both 2 cell populations. Interestingly, in late stage of differentiation, the WJMSCs-SUT2 had faster response to hepatic-lineage induction than WJMSCs-SUT1 did. This result was supported by the early expression of *HNF*4*α* transcripts on day 12 while it was undetectable in MSCDHCs from WJMSCs-SUT1 on the same day. However, both the WJMSCs-SUT1 and WJMSCs-SUT2 could express all tested hepatic-lineage markers at mRNA level at the end stage of differentiation period (Figure 3). 

**Figure 4:**
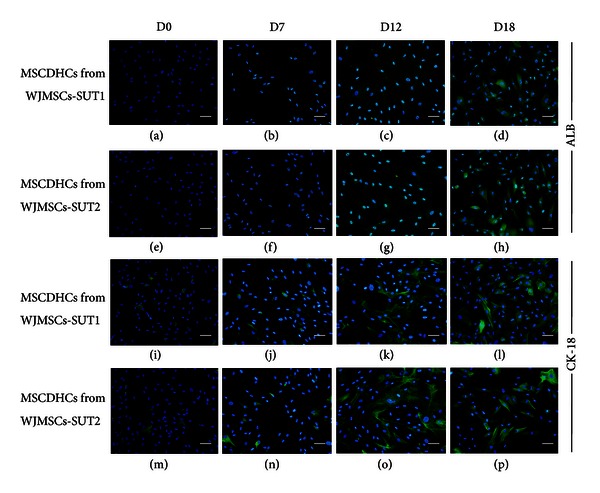
Hepatic-specific protein expressions of the differentiated cells after induction for 18 days. Albumin (ALB) expression analysis: MSCDHCs generated from WJMSCs-SUT1 ((a)–(d)) and WJMSCs-SUT2 ((e)–(h)) were stained with rabbit polyclonal anti-human albumin antibody following the differentiation period. For detection of cytokeratin 18 (CK-18) expression, MSCDHCs generated from WJMSCs-SUT1 ((i)–(l)) and WJMSCs-SUT2 ((m)–(p)) were stained with mouse monoclonal anti-human cytokeratin 18 antibody following the differentiation period. Cells nuclei were counterstained with DAPI. Original magnifications: ×200 for all pictures. Bars indicate 50 *μ*m.

We further analyzed more mature hepatic markers, albumin (ALB), and cytokeratin 18 (CK-18), at protein level. The low expression of CK-18 was observed in undifferentiated stage (day 0) of these 2 cell lines (Figures 4(i) and 4(m)). Following differentiation, more than 80% MSCDHCs generated from both 2 cell populations showed the significant upregulations of ALB (Figures 4(a)–4(h)) and CK-18 (Figures 4(i)–4(p)) in a time-dependent manner. These findings suggest that both WJMSCs-SUT1 and WJMSCs-SUT2 could also express hepatic-lineage markers at protein level in response to our induction medium in addition to the other hepatic mRNA expressions. Taken together, these results demonstrate that WJMSCs-SUT1 and WJMSCs-SUT2 could be induced into hepatocyte-like cells feature not only in a cellular phenotype aspect but also in hepatic marker expressions both at mRNA and protein levels. 

### 3.3. Hepatocytes Functions of MSCs-Derived Hepatocyte-Like Cells

**Figure 5:**
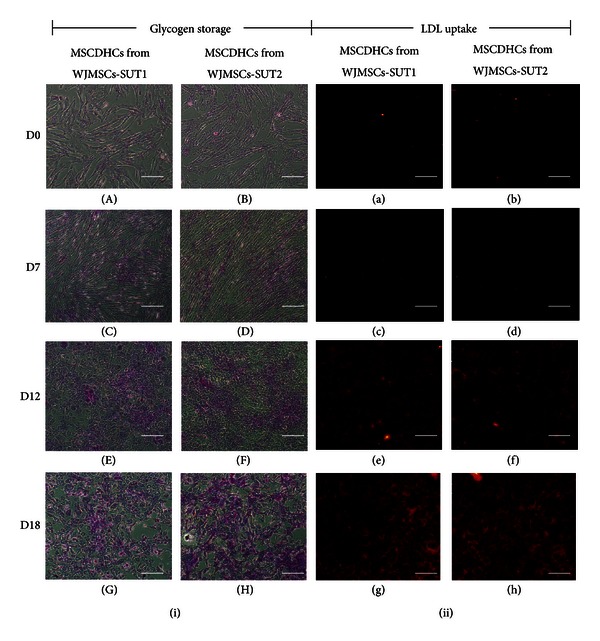
The capacities of the differentiated cells to store glycogen and accumulate LDL inside the cells after induction for 18 days. Glycogen storage of MSCDHCs generated from WJMSCs-SUT1 ((i): (A), (C), (E), and (G)) and WJMSCs-SUT2 ((i): (B), (D), (F), and (H)) were characterized by PAS staining following the differentiation period. LDL-uptake capacity of MSCDHCs generated from WJMSCs-SUT1 ((ii): (a), (c), (e), and (g)) and WJMSCs-SUT2 ((ii): (b), (d), (f), and (h)) were analyzed by LDL-uptake assay following the differentiation period. Original magnifications: ×100 for all pictures. Bars indicate 100 *μ*m.

**Figure 6:**
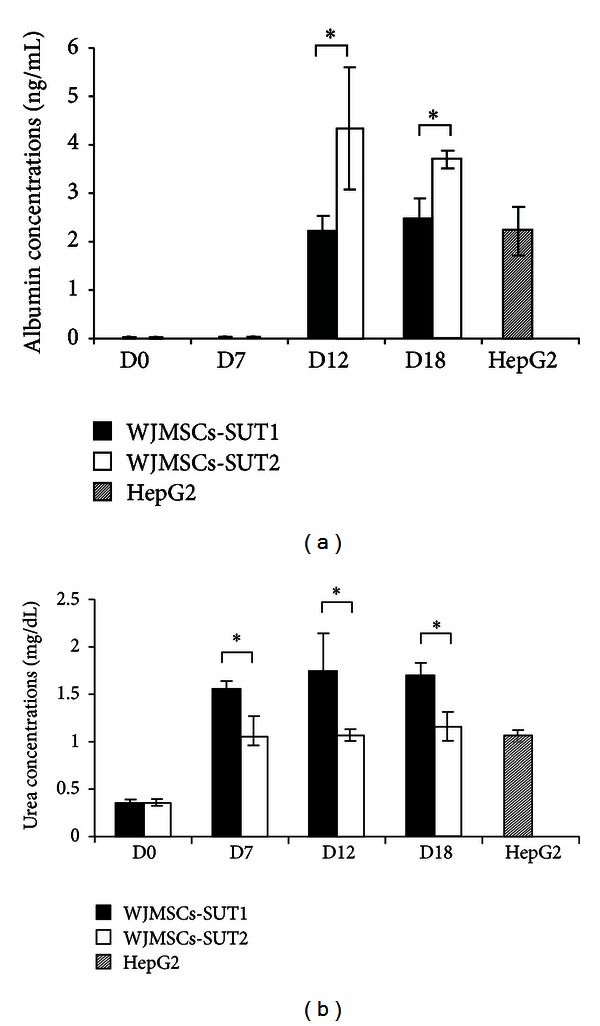
The capacities of the differentiated cells to secrete albumin and produce urea after induction for 18 days. Albumin secretion (a) and urea production (b) of MSCDHCs generated from WJMSCs-SUT1 and WJMSCs-SUT2 were determined by ELISA and colorimetric assay following the differentiation period, respectively. HepG2 was used as positive control. All data are presented as mean ±SD (*n* = 3). *P < 0.05.

The biological functions of hepatocytes were evaluated in the differentiated cells at various stages upon differentiation. First, the capacity of glycogen storage was analyzed by PAS staining. The results showed low level of PAS staining in undifferentiated stage (day 0) of WJMSCs-SUT1 and WJMSCs-SUT2. However, upon induction, MSCDHCs generated from both 2 cell lines displayed significant positive staining of glycogen granules in their cytoplasm as early as on day 7 and more stronger staining on day 12 and the strongest at day 18 (Figure 5(a)). In addition, more than 80% of the differentiated cells from these 2 cell populations had capacity to accumulate low-density lipoprotein (LDL) inside the cells like functional hepatocytes in a time-dependent manner whereas undifferentiated cells (day 0) did not perform this ability (Figure 5(b)). Furthermore, albumin secretion and urea production were also determined in order to confirm the hepatocytes functions. We found that undifferentiated stage (day 0) of WJMSCs-SUT1 and WJMSCs-SUT2 did not secrete detectable levels of albumin even though they have been detected albumin expression both at gene and protein levels. However, during differentiation processes, MSCDHCs generated from both 2 cell lines continuously secreted albumin since entry into maturation step from day 12 to day 18 (Figure 6(a)). Finally, we also tested metabolic function of the differentiated cells to detoxify ammonia to urea which is a less toxic form. In consistency, the differentiated cells from WJMSCs-SUT1 and WJMSCs-SUT2 could produce urea in response to ammonia detoxification process at all points of differentiation period (Figure 6(b)). Taken together, these data suggest that both WJMSCs-SUT1 and WJMSCs-SUT2 could commit toward functional hepatocyte-like cells by our new hepatogenic induction protocol under hypoxic condition. 

Furthermore, the percentage of differentiated cells has been evaluated based on both hepatic phenotypes (ALB-expressing cells, CK-18-expressing cells) and functions (LDL and PAS positive stained cells) by counting the numbers of positive stained cells of each marker against the total numbers of the cells as references by their stained cell nuclei from the same sample. For hepatic phenotypes, the induced cells highly expressed hepatic markers both ALB (89.00 ± 1.00% for WJMSCs-SUT1, 88.33 ± 1.53% for WJMSCs-SUT2) and CK-18 (85.00 ± 2.00% for WJMSCs-SUT1, 83.00 ± 2.00% for WJMSCs-SUT2). For hepatic functions, these cells could highly store glycogen inside the cells as shown by positive stained cells of PAS staining (82.33 ± 2.52% for WJMSCs-SUT1, 87.00 ± 2.00% for WJMSCs-SUT2) whereas more than 80% of LDL positive stained cells were observed in both 2 cell lines. Taken together, our developed protocol under hypoxic condition could generate high yield of functional hepatocytes ranging from 80 to 90% of the induced cells from both WJMSCs-SUT1 and WJMSCs-SUT2. Here, we successfully generated a new method to establish high yield and functional hepatocyte-like cells from WJ-MSCs which may be advantages in clinical applications, hepatic toxicity drug screening test including toxicological research, and other medical applications. 

## 4. Discussion

The WJ-MSCs are expected as a promising tool for therapeutic purposes. This is mainly due to their wide range differentiation capacity, noninvasive collection, and low immunogenicity. They have immunomodulatory as inhibitory effect on stimulated T cells* in vitro *that would be an advantage to allogenic transplantation [26]. Previous study found that human umbilical cord matrix stem cells could be successfully xenotransplanted into immunocompetent rats with little or no host immune response [27]. These findings support low immunogenic property of WJ-MSCs *in vitro*. In addition, WJ-MSCs have broader differentiation potential into several cell types such as insulin-producing cells [28], nerve cells [29], and cardiomyocytes [30] including hepatocyte-like cells [17]. Furthermore, preclinical studies found that WJ-MSCs could improve the liver functions, restore the survival rate, and decrease liver fibrosis in animal model with liver injury [13, 31]. Although WJ-MSCs have not been applied in clinical trials related to liver diseases, recent pilot study demonstrated the safety and successful treatment with placenta-derived MSCs in type 2 diabetes patients [32]. Based on their advantages, WJ-MSCs seem to possess beneficial effect on liver disease treatment. 

In this study, we aim to investigate the efficiency of a new established method for *in vitro* induction of WJ-MSCs into hepatic lineage by testing with our human WJ-MSCs cell lines as WJMSCs-SUT1 and WJMSCs-SUT2. Based on several hepatogenic induction methods, we developed a new two-step protocol to induce *in vitro* hepatogenic differentiation of WJ-MSCs under hypoxic condition which resembles the normal microenvironment in human physiology. Typically, the existing published protocols that have been used for hepatogenic induction of MSCs are performed in normal atmosphere of 20% O_2_ and 5% CO_2_. Here, we used hypoxic environment (5% O_2_ and 5% CO_2_) to accelerate hepatogenic differentiation potential of WJ-MSCs based on the superior advantages of this condition. In human ES cells, it has evidence that hypoxia is suitable for using in final hepatic induction to produce high yield of hepatocyte-like cells [33]. Moreover, hypoxia has been shown to activate hypoxia-inducible factor 1 (*HIF-1*) expression which has target gene asretinoic acid-receptor-related orphan receptor alpha (*Rorα*) transcripts in HepG2 [34]. The Rora upregulations are involved in controlling *ADIPOQ *and* G6PC* expressions which play roles in regulation of lipid and glucose metabolisms in hepatic cells, respectively [35]. From these data, hypoxia seems to provide appropriate environment for maintaining physiological activities including hepatic-lineage differentiation in liver cells. In addition, hypoxic preculturing of MSCs has been shown to increase cell homing, migration, and engraftment efficiency both* in vitro* and *in vivo* studies [36]. Therefore, it has possibility that hypoxic condition also enhances these efficiencies of MSCDHCs in addition to the improvement of the hepatogenic differentiation. The hepatocyte-like cells generated from hypoxic condition may retain these promising abilities which provide a great benefit in therapeutic purposes. 

According to developing a new protocol, the achievement of MSCDHCs generation was observed from both WJMSCs-SUT1 and WJMSCs-SUT2 by sequential treatment with our new cytokines-based cocktail medium. Sequential exposure of these cytokines was mimicked from the behavior of mouse embryonic liver development. In early stage of differentiation, we used FGF-4, HGF, and nicotinamide as main induction factors. In facts, FGFs expressed by cardiac mesoderm play a role in induction ventral foregut endoderm to initiate early liver development. Afterward, liver bud formation is occurred and rapid hepatoblast proliferation is stimulated by HGF that is a crucial factor for hepatogenesis. Nicotinamide is a water-soluble amide form of niacin or vitamin B3 which serves as primary precursor of nicotinamide adenine dinucleotide (NAD^+^) and the phosphorylated derivative NADP^+^ synthesis [37]. Actually, NAD^+^ and NADP^+^ play roles in maintaining energy for cellular functions including DNA repair and genomic stability. For hepatogenic differentiation, nicotinamide has been shown to enhance the proliferation of primary rat hepatocytes and formation of small hepatocytes colonies [38]. Based on these data, we also used nicotinamide in combination with our first stage hepatogenic induction medium. In late stage of differentiation, we utilized OSM, dexamethasone, and ITS as major factors. It has been reported that OSM secreted by hematopoietic cells is involved in the maturation fate of fetal hepatocytes [18]. In addition to these factors, it has been reported that dexamethasone is required for maintaining the expression of liver-enriched transcription factors which are essential for stimulating liver-specific genes transcription [39]. ITS is known as chemically defined supplement for supportive *in vitro* proliferation in various mammalian cells. For hepatogenic induction, ITS seems to maintain cell survival as monolayer during the induction period. Thus, we also used dexamethasone and ITS to induce more mature hepatocytes differentiation in our system. 

For hepatic-lineage marker expressions, *HNF*4*α* is a crucial liver-enriched transcription factor that plays an important role in stimulating the expression of liver-specific genes during liver development [40]. During the hepatogenic differentiation processes, early expression of *HNF*4*α* was detected in MSCDHCs generated from WJMSCs-SUT2 on day 12 which correlated with the upregulations of mature hepatic markers such as *ALB*, *AAT*, and *CYP*3*A*4. Similarly, MSCDHCs generated from WJMSCs-SUT1 showed the compatibility of *HNF*4*α* and hepatic-specific gene expressions but delayed to day 18. These findings imply that WJMSCs-SUT2 had a trend to differentiate into hepatic lineage in response to our induction method faster than WJMSCs-SUT1 did. Although these hepatocyte-like cells could express all mature hepatic markers, the expression of *AFP* which is a marker of immature hepatocytes was still detected even at late stage of differentiation. These data suggest that the hepatocyte-like cells or MSCDHCs generated from both 2 cell lines contain some hepatoblasts or hepatic progenitor cells. Similarly, previous studies demonstrated that human placenta-derived multipotent cells [41], umbilical cord matrix stem cells [17], and mesenchymal stromal cells derived from umbilical cord Wharton's jelly [10] were not yet fully differentiated into mature hepatocytes because *AFP* expression was still detected in the differentiated cells throughout the induction period. Based on these results, MSCs isolated from primitive origin seem to be reserved the immature phenotype even though they have driven toward specific lineage. 

In a late stage of differentiation, MSCDHCs generated from both WJMSCs-SUT1 and WJMSCs-SUT2 achieved characteristics of functional hepatocytes which were confirmed by LDL uptake assay, PAS staining for glycogen storage, albumin secretion, and urea assay. In comparison to previous published protocols [10, 17–21], our method provided high yield production of hepatocyte-like cells from WJ-MSCs, approximately more than 80% of the induced cells. Our protocol does not need any extracellular matrix substances for coating the culture dish to enhance hepatogenic differentiation. Moreover, this method required a shorter differentiation period, approximately 2 weeks, as compared to the other protocols which performed longer, at least for 3 weeks. Here, we demonstrate a simple, high efficiency, and time saving protocol for the generation of functional hepatocyte-like cells from WJ-MSCs. Altogether, these findings provide a new alternative option for an *in vitro* hepatocytes generation which may overcome the limitation of liver cell donors for future use in clinical applications. 

## 5. Conclusions

In summary, we report a simple, high efficiency, and time saving protocol for the generation of functional hepatocyte-like cells from WJ-MSCs. This method succeeds to drive WJ-MSCs into hepatic lineage under hypoxic condition. The achievement of hepatogenic differentiation was confirmed by both phenotypes and functions including hepatic marker expressions both at gene and protein levels, glycogen storage, low-density lipoprotein uptake, urea production, and albumin secretion. The achievement of MSCDHCs generation from this new method may provide greater potential in clinical applications and overcome the shortage of donor livers which is a major limitation of orthotopic liver transplantation. The implications of this work are therapeutic value in cell-based therapy for liver disease treatments and other regenerative medicine, hepatic toxicity drug screening in pharmacological approaches, and other medical applications in the future. 

## Figures and Tables

**Table 1:** List of primer sequences used in this study.

Gene	Primer sequence (5′ to 3′)	Annealing Temp.	Size (bp)	Reference
*AFP *	GCTTGGTGGTGGATGAAACA TCCTCTGTTATTTGTGGCTTTTG	62	157	[22]
*ALB *	TGAGAAAACGCCAGTAAGTGAC TGCGAAATCATCCATAACAGC	62	265	[22]
*CYP3A4 *	CCTTACAT TACACACCCTTTGGAAGT AGCTCAATGCATGTACAGAATCCCCGGTTA	62	382	[22]
*HNF4*α**	GCCTACCTCAAAGCCATCAT GACCCTCCCAGCAGCATCTC	62	275	[22]
*AAT *	ACATTTACCCAAACTGTCCATT GCTTCAGTCCCTTTCTCGTC	56	183	[23]
*TTR *	GGTGAATCCAAGTGTCCTCTGAT GTGACGACAGCCGTGGTGGAA	61	352	[24]
*G6P *	GCTGGAGTCCTGTCAGGCATTGC TAGAGCTGAGGCGGAATGGGAG	56	350	[23]
*TAT *	CCCCTGTGGGTCAGTGTT GTGCGACATAGGATGCTTTT	56	345	[23]
*GAPDH *	AGCCACATCGCTCAGACACC GTACTCAGCGGCCAGCATCG	60	302	[25]

*ALB*: albumin; *AFP*: alpha-fetoprotein; *AAT*: alpha-1-antitrypsin; *CYP3A4*: cytochrome P450 type 3A4; *GAPDH*: glyceraldehyde-3-phosphate dehydrogenase; *G6P*: glucose-6-phosphatase; *HNF4*α**: hepatocyte nuclear factor 4*α*; *TAT*: tyrosine aminotransferase; *TTR*: transthyretin; Temp.: temperature; bp: base pair.
